# Gemcitabine-based or capecitabine-based chemoradiotherapy for locally advanced pancreatic cancer (SCALOP): a multicentre, randomised, phase 2 trial

**DOI:** 10.1016/S1470-2045(13)70021-4

**Published:** 2013-04

**Authors:** Somnath Mukherjee, Christopher N Hurt, John Bridgewater, Stephen Falk, Sebastian Cummins, Harpreet Wasan, Tom Crosby, Catherine Jephcott, Rajarshi Roy, Ganesh Radhakrishna, Alec McDonald, Ruby Ray, George Joseph, John Staffurth, Ross A Abrams, Gareth Griffiths, Tim Maughan

**Affiliations:** a Gray Institute for Radiation Oncology and Biology, University of Oxford, NIHR Oxford Biomedical Research Centre, Oxford, UK; b Wales Cancer Trials Unit, Cardiff University, Cardiff, UK; c Institute of Cancer and Genetics, Cardiff University, Cardiff, UK; d UCL Cancer Institute, University College London, London, UK; e Bristol Haematology and Oncology Centre, Bristol, UK; f St Luke's Cancer Centre, Royal Surrey County Hospital, Guildford, UK; g Hammersmith Hospital, London, UK; h Velindre Cancer Centre, Velindre Hospital, Cardiff, UK; i Department of Oncology, Addenbrooke's Hospital, Cambridge, UK; j Diana Princess of Wales Hospital, Grimsby, UK; k St James's Institute of Oncology, St James's University Hospital, Leeds, UK; l Beatson West of Scotland Cancer Centre, Glasgow, UK; m Department of Radiation Oncology, Rush University Medical Center, Chicago, IL, USA; n Cardiff NCRI RTTQA Centre, Velindre NHS Trust, Cardiff, UK

## Abstract

**Background:**

In the UK, chemotherapy is the standard treatment for inoperable, locally advanced, non-metastatic pancreatic cancer. Chemoradiotherapy is also an acceptable treatment option, for which gemcitabine, fluorouracil, or capecitabine can be used as concurrent chemotherapy agents. We aimed to assess the activity, safety, and feasibility of both gemcitabine-based and capecitabine-based chemoradiotherapy after induction chemotherapy for patients with locally advanced pancreatic cancer.

**Methods:**

In this open-label, randomised, two-arm, phase 2 trial, patients aged 18 years or older with histologically proven, locally advanced pancreatic cancer (with a tumour diameter of 7 cm or less) were recruited from 28 UK centres between Dec 24, 2009 and Oct 25, 2011. After 12 weeks of induction gemcitabine and capecitabine chemotherapy (three cycles of gemcitabine [1000 mg/m^2^ on days 1, 8, 15 of a 28-day cycle] and capecitabine [830 mg/m^2^ twice daily on days 1–21 of a 28-day cycle]), patients with stable or responding disease, tumour diameter of 6 cm or less, and WHO performance status 0–1 were randomly assigned to receive a further cycle of gemcitabine and capecitabine chemotherapy followed by either gemcitabine (300 mg/m^2^ once per week) or capecitabine (830 mg/m^2^ twice daily, Monday to Friday only), both in combination with radiation (50·4 Gy in 28 fractions). Randomisation (1:1) was done via a central computerised system and used stratified minimisation. The primary endpoint was 9-month progression-free survival, analysed by intention to treat including only those patients with valid CT assessments. This trial is registered with ISRCTN, number 96169987.

**Findings:**

114 patients were registered and 74 were randomly allocated (38 to the gemcitabine group and 36 to the capecitabine group). After 9 months, 22 of 35 assessable patients (62·9%, 80% CI 50·6–73·9) in the capecitabine group and 18 of 35 assessable patients (51·4%, 39·4–63·4) in the gemcitabine group had not progressed. Median overall survival was 15·2 months (95% CI 13·9–19·2) in the capecitabine group and 13·4 months (95% CI 11·0–15·7) in the gemcitabine group (adjusted hazard ratio [HR] 0·39, 95% CI 0·18–0·81; p=0·012). 12-month overall survival was 79·2% (95% CI 61·1–89·5) in the capecitabine group and 64·2 (95% CI 46·4–77·5) in the gemcitabine group. Median progression-free survival was 12·0 months (95% CI 10·2–14·6) in the capecitabine group and 10·4 months (95% CI 8·9–12·5) in the gemcitabine group (adjusted HR 0·60, 95% CI 0·32–1·12; p=0·11). Eight patients in the capecitabine group had an objective response at 26 weeks, as did seven in the gemcitabine group. More patients in the gemcitabine group than in the capecitabine group had grade 3–4 haematological toxic effects (seven [18%] *vs* none, p=0·008) and non-haematological toxic effects (ten [26%] *vs* four [12%], p=0·12) during chemoradiation treatment; the most frequent events were leucopenia, neutropenia, and fatigue. Two patients in the capecitabine group progressed during the fourth cycle of induction chemotherapy. Of the 34 patients in the capecitabine group who received chemoradiotherapy, 25 (74%) received the full protocol dose of radiotherapy, compared with 26 (68%) of 38 patients in the gemcitabine group. Quality-of-life scores were not significantly different between the treatment groups.

**Interpretation:**

Our results suggest that a capecitabine-based regimen might be preferable to a gemcitabine-based regimen in the context of consolidation chemoradiotherapy after a course of induction chemotherapy for locally advanced pancreatic cancer. However, these findings should be interpreted with caution because the difference in the primary endpoint was non-significant and the number of patients in the trial was small.

**Funding:**

Cancer Research UK.

## Introduction

In 2010, 8463 new cases of pancreatic cancer were diagnosed in the UK and 7901 people died of the disease.^1^ At diagnosis, 30% of patients have locally advanced, inoperable disease.^2^ For these patients, chemotherapy alone or chemoradiotherapy are regarded as acceptable treatment options.^3^, ^4^, ^5^, ^6^ Randomised trials^7^, ^8^ that compared the two strategies have had conflicting results and therefore have not been able to define a preferred standard of care.

Investigators of several studies have proposed the use of induction chemotherapy to select appropriate patients who are most likely to benefit from chemoradiation treatment.^9^, ^10^, ^11^ This approach spares intensive local treatment for patients with chemotherapy-resistant or rapidly progressing systemic disease. Non-randomised studies that used this method of patient selection have reported overall survival of about 15–19 months.^9^, ^12^, ^13^, ^14^, ^15^, ^16^, ^17^ The international, randomised, phase 3 study LAP-07 (NCT00634725), which is comparing chemoradiotherapy with chemotherapy, is expected to be reported in 2013.

Both fluoropyrimidines and gemcitabine have been used concurrently with radiotherapy in patients with pancreatic cancer. Fluorouracil is most widely used, but gemcitabine radiosensitisation has been used in some studies because of its systemic activity in pancreatic cancer and potent radiosensitising properties.^3^, ^18^ Three randomised, controlled trials^19^, ^20^, ^21^ with small numbers of patients have compared fluorouracil with gemcitabine chemoradiotherapy as primary treatment for locally advanced pancreatic cancer; one^19^ showed a significant overall survival benefit for gemcitabine-based treatment, but the others^20^, ^21^ did not show a significant difference between the regimens. A meta-analysis of these data again suggested a survival advantage of gemcitabine compared with fluorouracil chemoradiotherapy, but at the cost of greater toxicity.^22^ These data are, however, not conclusive enough to define practice, because the trials had small numbers of patients (19–62 per trial). No study has compared the activity of gemcitabine-based chemoradiotherapy with a chemoradiotherapy regimen that uses the oral fluorouracil prodrug capecitabine for treatment of locally advanced pancreatic cancer.

We designed the SCALOP (Selective Chemoradiation in Advanced LOcalised Pancreatic Cancer) trial to assess the activity, safety, and feasibility of induction chemotherapy followed by chemoradiotherapy and to identify the relative benefits and toxicities of gemcitabine and capecitabine as concurrent chemotherapy agents.

## Methods

### Study design and patients

The SCALOP trial was a multicentre, open-label, randomised, parallel, two-arm, phase 2 trial. Patients aged 18 years or older were eligible if they had histologically or cytologically proven, locally advanced, non-metastatic, and inoperable (or operable but medically unfit for surgery) pancreatic cancer; a tumour diameter of 7 cm or less; WHO performance status 0–2; and adequate haematological, liver, and renal function (full inclusion and exclusion criteria are listed in the appendix). All potential patients were discussed at regional pancreatic multidisciplinary team meetings in the presence of specialist pancreatic surgeons and radiologists for decisions about inoperability, but the exact criteria for inoperability were left to the treating multidisciplinary team. Decisions with respect to patients deemed medically unfit for surgery were taken by the treating clinicians, on the basis of the patient's comorbidities and the team's opinion about whether or not they could withstand major pancreatic surgery.

Response after three cycles of induction gemcitabine and capecitabine chemotherapy was assessed with a CT scan. Patients were eligible for random allocation if they had responding or stable disease (according to RECIST criteria, version 1.1); a tumour diameter of 6 cm or less; WHO performance status 0–1; adequate haematological, liver, and renal function (as for registration); and no greater than 10% weight loss from baseline (defined as weight at registration).

All patients had to provide written informed consent before registration and the trial protocol was approved by the UK Medicines and Healthcare Products Regulatory Agency and a multicentre research ethics committee. The SCALOP trial was sponsored by Cardiff University and coordinated by the Wales Cancer Trials Unit (WCTU) at Cardiff University. The study protocol is available from the WCTU website.

### Randomisation and masking

After three cycles of induction chemotherapy, eligible patients were randomly assigned in a 1:1 ratio to receive either gemcitabine-based or capecitabine-based chemoradiotherapy, by use of the method of minimisation with a random element (80:20). Randomisation was stratified by recruiting hospital, WHO performance status (0 *vs* 1), and disease location (head *vs* body or tail). The research nurses who recruited the patients telephoned the WCTU, where randomisation was done on a computerised system by a trial or data manager. The study had an open-label design, so treatment allocation was not masked from patients or investigators.

### Procedures

Induction chemotherapy consisted of three cycles of gemcitabine (1000 mg/m^2^ intravenously for 1 h on days 1, 8, and 15 of a 28 day cycle) and capecitabine (830 mg/m^2^ orally, twice daily on days 1–21 of a 28 day cycle). Patients eligible for randomisation were allocated to receive a further cycle of gemcitabine and capecitabine chemotherapy at the same doses as were used during induction, followed by radiotherapy in combination with either gemcitabine (300 mg/m^2^ once per week, total six doses) or capecitabine (830 mg/m^2^ twice daily on days of radiotherapy [Monday to Friday only]) A total dose of 50·4 Gy in 28 daily fractions (Monday to Friday only) was delivered over 5·5 weeks by use of 3D conformal or intensity-modulated radiotherapy planning.

Dose modifications for gemcitabine were made on the basis of neutrophil and platelet counts on the day of (or day before) administration, as previously described.^23^ Capecitabine was withheld for grade 2 or higher non-haematological toxic effects until they resolved to grade 1; for recurrent grade 2 toxic effects, doses were sequentially reduced to 75% and then 50%. Dose reductions made during induction chemotherapy were maintained during the chemoradiotherapy phase. Chemoradiotherapy was discontinued if grade 3 or higher gastrointestinal toxic effects recurred more than once during chemoradiation treatment. Permitted dose reductions were predefined in the study protocol.

All patients underwent contrast-enhanced planning CT simulation with 200–300 mL water as oral contrast. Gross tumour volume consisted of the primary tumour and any node with short axis diameter of 1 cm or more. Planning target volume included the gross tumour volume with a margin of 2·0 cm in the craniocaudal direction and 1·5 cm in all other directions. Prophylactic irradiation of uninvolved regional nodes was not done. The mandated Radiotherapy Trials Quality Assurance (RTTQA) programme consisted of a detailed radiotherapy protocol developed by the trial management group and the Cardiff NCRI RTTQA Centre; a radiotherapy atlas that consisted of two cases (one pancreatic head tumour and one pancreatic body tumour) with the gross tumour volume outlined as per the RTTQA radiotherapy protocol; and a pretrial test case (inoperable carcinoma, head of pancreas). Gross tumour volume had to be outlined by the lead investigator and gastrointestinal radiologist from each centre and radiotherapy planned as per the trial protocol. The tumour outlines and the radiotherapy plans were assessed centrally and fed back to investigators. On-trial RTTQA consisted of a planning assessment form, which was completed during planning for each patient and reviewed centrally before the start of radiotherapy.

Assessments in the treatment period and during follow-up consisted of medical history, physical examination, and assessment of WHO performance status and toxic effects. Capecitabine compliance was assessed by counting the number of tablets at each visit. Laboratory tests included haematological and biochemical tests, including tumour marker (CA19-9) assessments at stipulated timepoints (at registration and at 17, 26, 39, and 52 weeks). Contrast-enhanced CT scans of thorax and abdomen to assess response were done at baseline (registration), at 13 weeks (before randomisation), and at weeks 26, 39, and 52. RECIST (version 1.1) criteria were used for response reporting. Patients were clinically reviewed (including full blood counts and serum renal and liver profiles) every 4 weeks during induction chemotherapy, every week during chemoradiotherapy, and every 12 weeks thereafter, until the final follow-up visit at week 52. At each visit, toxic effects were reported as per Common Terminology Criteria of Adverse Events (CTCAE, version 3.0). After chemoradiotherapy, all patients were discussed by the regional multidisciplinary team and were reassessed for operability, with suitable patients taken to surgery. Those not suitable for surgery were kept on trial follow-up and additional chemotherapy was not recommended unless disease progression was seen. Progression was defined according to radiological criteria, and an isolated rise in CA19-9 was not regarded as a criterion for progression. Treatment after progression was as per institutional practice, and no specific regimen was recommended.

Subsequent management beyond the trial follow-up period was at the discretion of the treating physician, but outcome data (death and disease progression) were collected every 3 months for those patients who had completed the 52-week assessment until the last patient had completed this assessment. Quality-of-life questionnaires (EORTC QLQ-C30^24^ and PAN26) were done at registration, week 17 (after induction chemotherapy), week 23 (immediately after chemoradiotherapy), and at weeks 26, 39, and 52. PAN26 results are not reported here; a detailed quality-of-life anaysis is planned, which will include these results.

### Statistical analysis

The primary endpoint of the trial was progression-free survival at 9 months. Progression-free survival was chosen in preference to overall survival because, on the basis of the study by Huguet and colleagues,^9^ we anticipated that activity could be detected earlier and with fewer patients.

A Fleming's single-stage design was assigned to each of the treatment groups to show worthwhile efficacy in each group separately—ie, the study was not formally powered to compare 9-month progression-free survival between the groups. We judged that progression-free survival at 9 months after registration of less than 30% would not be sufficiently large to warrant further investigation in a phase 3 setting, but that 50% or higher would warrant further investigation. Using Fleming's single-stage design (p1=0·30 and p2=0·50, setting α=0·1 [1-sided], 90% power), 38 participants needed to be allocated to each arm. We estimated that 25% of participants would either withdraw or not be eligible for random allocation at 13 weeks after registration, and that a minimum of 102 participants would therefore need to be recruited. Secondary endpoints were median and 12-month overall survival, median (including local and distant) progression-free survival, toxic effects (as per CTCAE, version 3.0), objective disease response, treatment compliance, and quality of life.

Analysis was done with the Stata 11 statistical package. The Clopper-Pearson exact binomial method was used to calculate 80% CIs for the primary endpoint of 9-month progression-free survival. Survival times were calculated from date of registration to that when an event occurred (ie, progression according to RECIST criteria or any death for progression-free survival, and any death for overall survival). Event-free patients were censored at the time last seen. Event time distributions were estimated by the Kaplan–Meier method and compared by use of an unadjusted log-rank test and hazard ratios (HRs) from Cox regression, both unadjusted and adjusted for randomisation stratification factors (the proportional hazards assumption was tested by use of Cox-Snell residuals and Schoenfeld's global test). Local progression-free survival was defined as time to progression without metastases or death by any cause. Distant progression-free survival was defined as time to progression with metastases or death by any cause. Efficacy endpoints were analysed according to the intention-to-treat principle—ie, all randomised patients who met the eligibility criteria were included in the analysis of their allocated group—whereas the analysis of toxic effects was restricted to patients who received at least 1 week of treatment. Objective disease response and 9-month progression-free survival were only assessed in those patients with valid CT assessments, defined as having been done within 4 weeks of the timepoints stipulated in the protocol (weeks 26, 39, and 52). Toxicity was assessed by comparing proportions of haematological and non-haematological toxic effects during chemoradiotherapy between treatment groups with Pearson's χ^2^ tests. Quality-of-life scores and CA19-9 concentrations were not normally distributed so were compared with Wilcoxon rank-sum tests. All analyses were prespecified in a statistical analysis plan.

This trial is registered at ISRCTN, number 96169987.

### Role of the funding source

Cancer Research UK's clinical trials awards and advisory committee approved the trial design. Cancer Research UK had no role in study design, data collection, analysis or interpretation, or writing of the report. The statistician (CNH) had full access to all of the data, and he and the corresponding author (SM) had the final responsibility to submit for publication.

## Results

**Figure 1:**
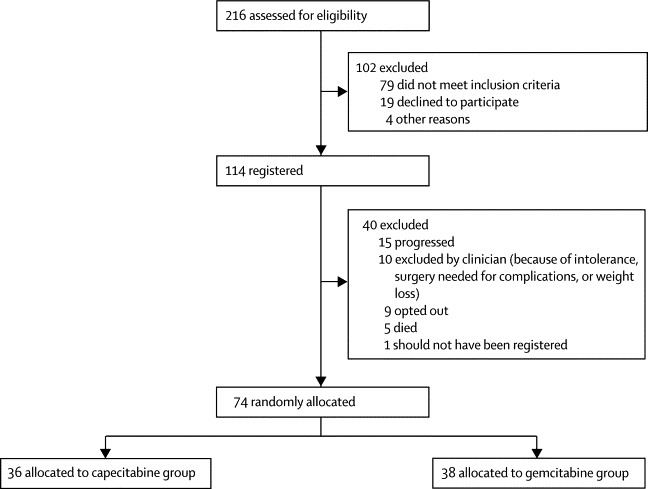
Trial profile

Between Dec 24, 2009, and Oct 25, 2011, 114 patients were registered into the trial from 28 hospitals across the UK. All patients were followed until progression, death, or 12-month follow-up assessment. 74 patients were eligible for randomisation after three cycles of induction chemotherapy; 38 were allocated to receive gemcitabine-based chemoradiation and 36 to receive capecitabine-based chemoradiation (figure 1).

The baseline characteristics of age, disease location, and WHO performance status were well balanced between the groups, but the gemcitabine group had a higher proportion of men than did the capecitabine group (table 1). The median number of days from staging to registration and from registration to start of chemotherapy was low in both groups; additionally, the median time from registration to start of radiotherapy was the same in both groups (table 1). On-trial review of planning assessment forms showed a 100% compliance with the radiotherapy protocol. A separate detailed analysis of the radiotherapy quality assurance process and radiotherapy compliance is planned.

**Table 1:** Patient characteristics

		**Capecitabine group (n=36)**	**Gemcitabine group (n=38)**	**Patients excluded at randomisation (n=40)**
**At enrolment (week 0)**				
Age (years)		63·1 (56·5–70·2)	66·0 (57·7–70·3)	64·2 (59·4–70·0)
Sex				
	Male	17 (47%)	24 (63%)	22 (55%)
	Female	19 (53%)	14 (37%)	18 (45%)
WHO performance status				
	0	20 (56%)	20 (53%)	15 (38%)
	1	14 (39%)	17 (45%)	20 (50%)
	2	2 (6%)	1 (3%)	5 (13%)
Mean estimated longest diameter of primary lesion (SD), cm		4·0 (1·2)	4·0 (1·5)	4·5 (1·6)
	Missing data	0	2 (5%)	1 (3%)
Number of days from staging CT scan to registration		7·0 (5·0–15·0)	5·0 (2·0–9·0)	10·0 (5·0–17·0)
Number of days from registration to start of chemotherapy		3·0 (1·0–5·0)	1·5 (0–4·0)	3·0 (1·0–6·0)
CA19-9 concentration (U/mL)		160 (27–710)	237 (110–971)	623 (95–1885)
Missing data		4 (11%)	3 (8%)	1 (3%)
**At randomisation (week 13)**				
WHO performance status				
	0	15 (42%)	16 (42%)	..
	1	21 (58%)	22 (58%)	..
Site of pancreatic tumour				
	Head	31 (86%)	32 (84%)	..
	Body or tail	5 (14%)	6 (16%)	..
Number of days from registration to start of chemoradiotherapy		118·5 (115–125)	118·5 (116–123)	..
	Progressed before start of chemoradiotherapy	2 (6%)	0	..

Data are n (%) or median (IQR), unless otherwise stated.

Table 2 shows treatment compliance during chemoradiotherapy. Two patients in the capecitabine group progressed before chemoradiation treatment could be started. Radiotherapy was well tolerated, with a slightly higher proportion of patients receiving at least 25 out of 28 fractions in the capecitabine group than in the gemcitabine group. A slightly higher proportion of patients in the gemcitabine group had 4 weeks or more of concurrent chemotherapy prescribed at 100% of the protocol dose. However, fewer patients in the capecitabine group needed dose reductions or stopped chemotherapy early than did those in the gemcitabine group.

**Table 2:** Treatment compliance during chemoradiotherapy

			Capecitabine group (n=34)*	**Gemcitabine group (n=38)**
**Chemotherapy**				
No dose reductions			23 (68%)	19 (50%)
Completed at least four weeks of chemotherapy at 100% dose			26 (76%)	30 (79%)
Stopped chemotherapy before 6 weeks			4 (12%)	10 (26%)
	Reasons for stopping			
		Haematological effects	0	5 (13%)
		Fatigue	0	2 (5%)
		Liver function	2 (6%)	0
		Diarrhoea	1 (3%)	0
		Hand-foot syndrome	1 (3%)	1 (3%)
		Sepsis	0	1 (3%)
		Patient request	0	1 (3%)
**Radiotherapy**				
Full protocol dose			25 (74%)	26 (68%)
25–27 fractions given			7 (21%)	8 (21%)
20–24 fractions given			0	3 (8%)
1–19 fractions given			2 (6%)	1 (3%)
No radiotherapy given			2 (6%)	0
Median dose Gy (IQR)			50·4 (48·6–50·4)	50·4 (48·6–50·4)

Data are n (%), unless otherwise stated.

* Two patients in the capecitabine group are excluded because they progressed during the fourth cycle of induction chemotherapy before any chemoradiation treatment could be given.

The main grade 3–4 toxic effects that occurred during chemoradiotherapy are summarised in table 3. More patients in the gemcitabine group than in the capecitabine group had any grade 3 or 4 toxic effects during induction chemotherapy (24 [67%] of 36 patients *vs* 17 [45%] of 38). During chemoradiotherapy, both regimens were well tolerated, but a smaller proportion of patients in the capecitabine group had haematological and non-haematological grade 3 or 4 toxic effects (haematological: none *vs* seven [18%] of 38 patients; χ^2^=6·94, p=0·008; non-haematological: four [12%] of 34 patients *vs* ten [26%] of 38 patients; χ^2^=2·43, p=0·12). No treatment-related deaths occurred.

**Table 3:** Grade 3–4 toxic effects

			**Induction chemotherapy (all randomised patients, n=74)**	**Chemoradiotherapy**	
				Capecitabine group (n=34)*	Gemcitabine group (n=38)
Any grade 3–4 effects			41 (55%)	4 (12%)	14 (37%)
Haematological			28 (38%)	0	7 (18%)
	Haemoglobin		2 (3%)	0	0
	Leucocytes		10 (14%)	0	5 (13%)
	Absolute neutrophil count		28 (38%)	0	4 (11%)
	Platelets		3 (4%)	0	1 (3%)
	Lymphocytes		0	0	1 (3%)
Non-haematological			24 (32%)	4 (12%)	10 (26%)
	Constitutional symptoms		2 (3%)	2 (6%)	5 (13%)
		Fatigue	2 (3%)	2 (6%)	4 (11%)
		Weight loss	0	0	1 (3%)
	Dermatological symptoms		6 (8%)	0	0
		Hand-foot syndrome	4 (5%)	0	0
		Other	2 (3%)	0	0
	Gastrointestinal symptoms		7 (9%)	0	6 (16%)
		Diarrhoea	3 (4%)	0	3 (8%)
		Nausea or vomiting	1 (1%)	0	3 (8%)
		Anorexia	0	0	3 (8%)
		Other	4 (5%)	0	0
	Infection		8 (11%)	0	2 (5%)
		Febrile neutropenia	2 (3%)	0	1 (3%)
		Infection with normal neutrophil count	7 (9%)	0	1 (3%)
	Vascular		3 (4%)	0	0
		Thrombosis, thrombus, or embolism	2 (3%)	0	0
		Other	1 (1%)	0	0
	Metabolic (laboratory)		10 (14%)	1 (3%)	3 (8%)
		Liver related	5 (7%)	0	1 (3%)
		Other	7 (9%)	1 (3%)	2 (5%)
	Other		4 (5%)	2 (6%)	3 (8%)

Data are n (%).

* Two patients in the capecitabine group are excluded because they progressed during the fourth cycle of induction chemotherapy before any chemoradiation treatment could be given.

The primary endpoint of 9-month progression-free survival was assessable in 35 patients from each group (in the gemcitabine group, one patient was too ill for CT scan, one refused, and one died before the scan was due; in the capecitabine group, one patient had a CT scan done more than 4 weeks after the 9-month timepoint). 22 patients (62·9%, 80% CI 50·6–73·9) in the capecitabine group had not progressed at 9 months, compared with 18 patients (51·4%, 39·4–63·4) in the gemcitabine group.

Table 4 shows the objective disease response at 26 weeks, 4 weeks after completion of chemoradiotherapy (when response conventionally reassessed). Five patients (two from the capecitabine group and three from the gemcitabine group) underwent resection of the primary tumour after completion of study treatment—four of these patients (two from each group) remained disease free at 52-week follow-up and one (from the gemcitabine group) died from postoperative complications (appendix). Full histological assessments were done for all of the resected specimens and were reported as ypT1N0 (n=1), ypT2N0 (n=1), and ypT3N0 (n=3); all had pathologically clear margins.

**Table 4:** Objective disease response at 26 weeks from registration

	Capecitabine group (n=35*)	**Gemcitabine group (n=36**†**)**
Complete response	2 (6%)	0
Partial response	6 (17%)	7 (19%)
Stable disease	22 (63%)	24 (67%)
Progressive disease	5 (14%)	5 (14%)

Response was determined according to RECIST (version 1.1) criteria. Data are n (%).

* CT scan not done or invalid (ie, more than 4 weeks either side of 26 weeks) for one patient.

† CT scan not done or invalid (ie, more than 4 weeks either side of 26 weeks) for two patients.

**Figure 2:**
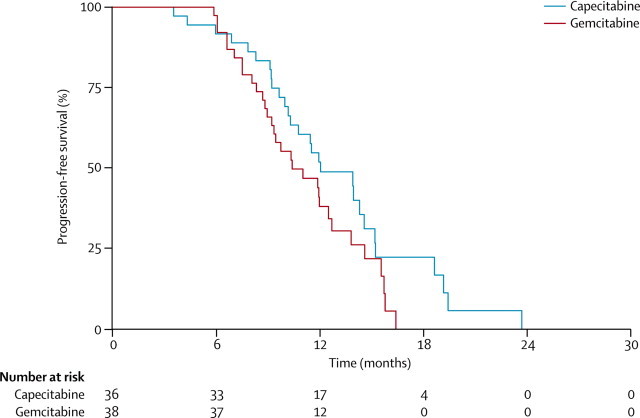
Kaplan–Meier estimates of progression-free survival, by treatment group

Median progression-free survival was 12·0 months (95% CI 10·2–14·6) in the capecitabine group and 10·4 months (95% CI 8·9–12·5) in the gemcitabine group (unadjusted HR 0·64, 95% CI 0·37–1·09; log-rank p=0·102; adjusted HR 0·60, 95% CI 0·32–1·12; p=0·11; figure 2). Median local progression-free survival was 14·6 months (95% CI 11·3–18·6) in the capecitabine group and 12·0 months (9·8–14·0) in the gemcitabine group. Distant progression-free survival was 14·3 months (95% CI 11·5–15·2) in the capecitabine group and 11·9 months (9·7–13·8) in the gemcitabine group. The pattern of disease progression at 12 months from registration is shown in table 5.

**Table 5:** Patterns of disease progression at 12 months from registration

		**Capecitabine group (n=36)**	**Gemcitabine group (n=38)**
Alive with no progression		8 (22%)	7 (18%)
Died before progression		7 (19%)	9 (24%)
Disease progressed		21 (58%)	22 (58%)
	Local	7 (33%)*	7 (32%)*
	Metastatic	11 (52%)*	10 (45%)*
	Both	3 (14%)*	5 (23%)*

Data are n (%).

* Percentages are of number of patients whose cancer had progressed.

**Figure 3:**
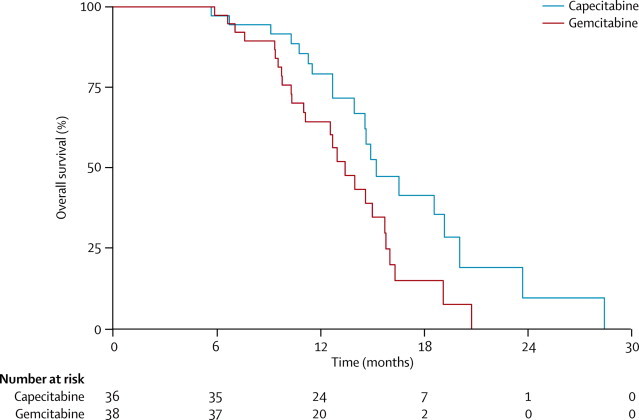
Kaplan–Meier estimates of overall survival, by treatment group

Median overall survival for all registered patients (n=114) was 12·7 months (95% CI 11·0–14·5); 12-month overall survival was 52·9% (95% CI 42·9–61·9). Patients who proceeded to be randomised to chemoradiotherapy (n=74) had a median overall survival of 14·6 months (95% CI 13·0–15·8) and 12-month survival of 77·5% (95% CI 65·8–85·6); patients who did not proceed to randomisation (n=40) had a median overall survival of 8·1 months (95% CI 4·1–10·5) and 12-month survival of 16·9% (95% CI 6·9–30·7). Median overall survival was 15·2 months (95% CI 13·9–19·2) in the capecitabine group and 13·4 months (95% CI 11·0–15·7) in the gemcitabine group (unadjusted HR 0·50, 95% CI 0·27–0·93; log-rank p=0·025; adjusted HR 0·39, 95% CI 0·18–0·81; p=0·012; figure 3). This difference was maintained in a sensitivity analysis that included sex and CA19-9 concentration (imbalanced at randomisation) in the adjusted Cox regression (p=0·003). 12-month overall survival was 79·2% (95% CI 61·1–89·5) in the capecitabine group and 64·2% (95% CI 46·4–77·5) in the gemcitabine group.

**Figure 4:**
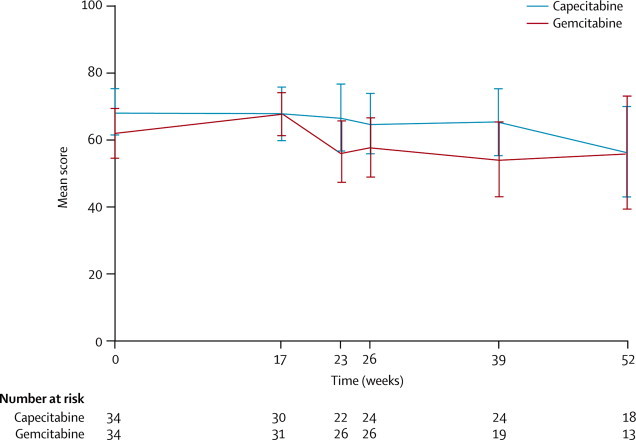
Quality of life scores Scores are from the QLQ-C30 questionnaire. Error bars show 95% CIs.

We assessed quality of life with the QLQ-C30 questionnaire (figure 4, appendix). Despite an apparent difference between the treatment groups, the scores at week 23 (immediately after completion of chemoradiotherapy) were not significantly different (z=–1·492; p=0·14; n=48). Nor was the difference between the changes in score from week 17 (before chemoradiation treatment) to week 23 significant (z=–1·514; p=0·13; n=45). A detailed quality-of-life analysis is planned.

No significant difference in CA19-9 concentrations was detected between the capecitabine group and the gemcitabine group, either at baseline (table 1; z=1·500; p=0·13; n=67) or at week 17, immediately before chemoradiotherapy (capecitabine median 29 U/mL (17–170); gemcitabine median 130 U/mL (21–251); z=1·603; p=0·11; n=58), in randomised patients. We divided all patients into groups on the basis of whether or not their CA19-9 concentrations at baseline and week 17 were lower than the median, and compared overall survival (table 6). Higher baseline CA19-9 was predictive of worse survival in both all recruited and all randomised patients (table 6). Higher week 17 CA19-9 concentrations were highly predictive of worse survival in randomised patients, but the fall in CA19-9 between baseline and week 17 was not. The significance of these results was maintained in multivariate models (table 6). The hazard ratio for overall survival between the capecitabine and gemcitabine groups is more significant in patients with high CA19-9 at baseline (HR 0·30, 95% CI 0·11–0·79; p=0·014) than in those with low baseline CA19-9 (0·55, 0·17–1·82; p=0·33). CA19-9 measurements at week 17 and 26 (ie, after chemoradiotherapy) were available for only 43 (58%) of 74 patients, which limits any further in-depth analysis.

**Table 6:** Overall survival by CA19-9 concentration at baseline and week 17

	**n**	**Median CA19-9 (IQR), U/mL**	**Overall survival in months (95% CI)**		**Univariate**		Multivariate*	
			CA19-9 less than median	CA19-9 median or higher	Hazard ratio (95% CI)	p value	Hazard ratio (95% CI)	p value
Baseline in all patients	106	331·5 (75–1074)	15·7 (12·9–16·5) (n=53)	10·4 (9·3–11·7) (n=53)	2·49 (1·53–4·03)	<0·001	2·53 (1·53–4·20)	<0·001
Baseline in randomised patients	67	212 (71–829)	16·3 (15·2–20·7) (n=33)	11·3 (9·7–13·4) (n=34)	4·29 (2·14–8·59)	<0·001	4·19 (2·09–8·40)	<0·001
Week 17 in randomised patients	58	44 (19–208)	16·3 (13·9–19·2) (n=29)	12·6 (10·3–14·0) (n=29)	3·37 (1·67–6·81)	0·001	3·84 (1·67–8·80)	0·002
Percentage fall in CA19-9 between baseline and week 17	54	−74·8% (−89·0 to −53·2)	14·6 (10·3–19·2) (n=27)	14·0 (12·7–16·0) (n=27)	1·26 (0·63–2·56)	0·51	1·12 (0·51–2·44)	0·780

* For all patients variables include sex, age, and WHO performance status; for randomised patients disease location is also included (centre could not be used because of small numbers).

## Discussion

Our results show that induction chemotherapy followed by gemcitabine-based or capecitabine-based chemoradiotherapy are both active regimens and can be given safely. The capecitabine-based regimen seems to have better safety and efficacy outcomes than the gemcitabine-based regimen, although the difference in progression-free survival at 9 months was not significant (panel).

Panel **Research in context**

**Systemic review**

We searched PubMed for research articles published in English before January 15, 2013, using the keywords “locally advanced pancreatic cancer” and “chemoradiotherapy”, and identified 179 articles. No randomised controlled trials or retrospective studies compared gemcitabine-based with capecitabine-based chemoradiotherapy. We identified two randomised trials^19^, ^21^ that compared gemcitabine-based with fluorouracil-based chemoradiotherapy and one^25^ that compared gemcitabine-based with paclitaxel-based chemoradiotherapy. We also identified one meta-analysis^22^ that compared gemcitabine-based with fluorouracil-based chemoradiotherapy. This meta-analysis included one additional randomised trial^26^ that was not identified in the original PubMed search, and therefore included three randomised trials and one retrospective comparative study,^20^ with a total of 229 patients. The results showed an overall survival advantage for gemcitabine compared with fluorouracil at 12 months from randomisation (risk ratio 1·54, 95% CI 1·05–2·26, p=0·03). Severe haematological toxic effects were more frequent in the gemcitabine group.

**Interpretation**

The higher toxicity of gemcitabine compared with the fluorouracil prodrug capecitabine seen in our study is consistent with previous research. However, by contrast with previous evidence, our results seem to show a survival advantage from capecitabine-based compared with gemcitabine-based chemoradiotherapy. Although these data should be interpreted with caution, because the difference in the primary endpoint was non-significant and the number of patients small, in the setting of induction chemotherapy, capecitabine-based chemoradiotherapy is safe and effective and might be preferable to gemcitabine-based chemoradiotherapy for patients with locally advanced pancreatic cancer.

Locally advanced pancreatic cancer has a poor prognosis and treatment advances have evolved slowly. The contribution of radiotherapy to the improvement of survival and quality of life is a controversial issue, since many patients die from rapidly emerging metastatic disease. Survival of patients with locally advanced pancreatic cancer who are treated with chemotherapy alone, extracted from studies that combine patients with locally advanced pancreatic cancer and those with metastatic disease, ranges from 9·9 to 10·3 months.^5^, ^23^ Chemoradiotherapy for inoperable adenocarcinoma of the pancreas has been a standard treatment in the USA since a series of seminal studies from the Gastrointestinal Studies Group in 1981.^6^ The relative benefit attributed to chemoradiotherapy compared with radiotherapy alone could be due in part to the extended administration of maintenance chemotherapy after chemoradiation in combined modality trial groups, which suggests that additional systemic therapy might confer advantage. This strategy has not been widely used in the UK, partly because of logistical issues, but also because of a paucity of supportive evidence and the perceived toxicity of chemoradiotherapy in patients with such a poor outlook.

Trials in the past 6–7 years that have assessed chemoradiotherapy have started with induction chemotherapy, with chemoradiotherapy given as a consolidation regimen for patients who do not develop metastases (typically 65–70% of patients). This approach enables the initiation of effective, full-dose systemic therapy immediately after diagnosis, followed by intensified treatment at the primary site for optimum tumour control and, in some cases, downstaging to enable surgical resection. Non-randomised trials that have used this approach have reported survival outcomes of 14–19 months in this select group of patients,^9^, ^13^, ^14^, ^15^, ^16^, ^17^, ^20^ and the results of one non-randomised study suggest that in patients with responding disease, this approach of switching to chemoradiotherapy could have better outcome than continuation of chemotherapy alone (overall survival of 15·0 *vs* 11·7 months, p=0·0009).^9^ A prospective, phase 3 trial (LAP-07) to assess the additional benefit of chemoradiotherapy in this setting has completed accrual in Europe. In this trial, after 3 months of induction chemotherapy, responding patients have been randomly allocated to either continue the same chemotherapy regimen or to switch to a capecitabine-based chemoradiotherapy regimen similar to that used for the capecitabine group in our study.

Our results suggest that the overall survival with induction chemotherapy followed by consolidation chemoradiotherapy seen here in a multicentre setting accords with the results of previous single-centre studies. 40 registered patients did not receive chemoradiotherapy because of death (n=5), poor general condition (n=10), progressive disease or ineligibility (n=16), or patient choice (n=9); in these patients median survival was only 8·1 months (95% CI 4·1–10·1). However, the overall median survival of all registered patients was 12·7 months (95% 11·0–14·5), which is better than previous outcomes in chemotherapy-only studies (roughly 10 months).^4^, ^5^

Grade 3–4 toxic effects were less frequent during consolidation chemoradiotherapy than during induction chemotherapy. The selection of fitter patients through induction treatment, limited-field radiotherapy, conformal radiation techniques, and above all, the prospective RTTQA programme will all have contributed to this favourable outcome. Moreover, because all patients received induction chemotherapy that used gemcitabine and capecitabine, potential chemotherapy-related toxic effects were identified and appropriate measures taken, including dose reduction instituted before the start of chemoradiotherapy.

Of the previous studies that compared gemcitabine-based and fluorouracil-based chemoradiotherapy,^19^, ^20^, ^21^ at least one study^19^ and a meta-analysis^22^ suggested better survival outcomes with gemcitabine. However these studies used chemoradiotherapy without induction chemotherapy and were done in unselected patients. Our results showed a significant advantage for the secondary endpoint of overall survival in the capecitabine group compared with the gemcitabine group, and the difference is clinically relevant, especially because of the apparently lower toxicity. Both distant and local progression-free survival seemed to be better in the capecitabine group as well, but the differences in progression-free survival were not significant.

The rationale behind the previous studies that compared gemcitabine with fluorouracil in a chemoradiotherapy setting was based on the hypothesis that gemcitabine is a more potent radiosensitiser than fluorouracil; however, in the clinical setting differentiating between the radiosensitising effect and the systemic contribution of the chemotherapy is difficult. The size of benefit from capecitabine in our study was similar for both distant and local progression-free survival, which might suggest that the systemic effect accounts for the difference.

An optimum concurrent dose of gemcitabine in conjunction with radiotherapy has not been defined. Gemcitabine is a potent radiosensitiser and increased doses of gemcitabine in combination with radiotherapy have been associated with heightened toxicity. In phase 1–2 clinical trials, once-per-week doses of gemcitabine have ranged from 250 mg/m^2^ to 1000 mg/m^2^, and the only phase 3 trial^7^ to use gemcitabine as a radiosensitiser used a dose of 600 mg/m^2^. That trial,^7^ in which patients were randomly assigned to receive either gemcitabine alone or gemcitabine with radiotherapy (50·4 Gy in 28 fractions), was stopped early because of poor recruitment, with 71 patients entered from eight US centres. Survival was slightly better in the chemoradiotherapy group than in the chemotherapy-only group (11·1 *vs* 9·2 months, p=0·017), but at the expense of increased grade 4–5 toxic effects (41% *vs* 9%). Phase 1–2 studies that used full-dose gemcitabine in combination with radical doses of radiotherapy have also been reported.^27^, ^28^ Although outcomes from these studies are promising and the toxicities are acceptable, the trials were done in a small number of experienced centres, and the results might not be reproducible in a large multicentre setting.

In our study we used a fairly low dose of gemcitabine (300 mg/m^2^ once per week). This reduced dose could possibly account for the difference in survival outcomes between the gemcitabine and capecitabine groups, since the reduction in dose of systemic therapy in the gemcitabine group might have compromised the outcome, whereas adequate systemic therapy was maintained in the capecitabine group. We can speculate that increasing the dose of gemcitabine chemoradiotherapy might have improved the survival outcomes in this group, but doing so would also have increased the frequency of grade 3–4 toxic effects.

The suggestion that the difference in outcomes between the treatment groups in our study could have been caused by a reduction in systemic therapy in the gemcitabine group would be consistent with evidence that systemic therapy is important in the management of locally advanced pancreatic cancer, as was shown by Chauffert and colleagues,^7^ in whose study compromise in the systemic adjuvant chemotherapy after primary fluorouracil and cisplatin-based chemoradiotherapy resulted in a poor outcome compared with chemotherapy alone. Additionally, in a retrospective series^29^ of 85 patients that compared outcomes in patients who received adjuvant chemotherapy after primary gemcitabine-based chemoradiotherapy with those who did not, 2-year overall survival was significantly better in those who received the adjuvant treatment (31·8% *vs* 12·4%, p<0·001). Neither our study nor LAP-07 recommended continuation of chemotherapy after chemoradiotherapy, because chemotherapy was given upfront in both. Whether recommencement of chemotherapy after chemoradiotherapy would provide additional benefit remains unknown. Nevertheless, in our trial, durable disease control was achieved with a median progression-free survival of 12 months in the capecitabine-based chemoradiation group. This finding suggests that in appropriately selected patients chemoradiotherapy can effectively delay tumour progression, even if chemotherapy is not recommenced. The roles of newer chemotherapy regimens such as FOLFIRINOX or of the continuation of further systemic treatment after chemoradiotherapy remain to be tested in future randomised studies.

Our study had several limitations. Although our results show a clinically relevant difference in overall survival between capecitabine-based and gemcitabine-based chemoradiotherapy, this finding should be interpreted cautiously since this outcome was not the primary endpoint and the number of patients included in the trial was small. Data for second-line treatment after disease progression were not obtained and an imbalance between the groups in terms of the proportion of patients who received second-line treatment might have affected the survival outcomes. Moreover, although randomised, the Fleming's design is not ideal for a direct comparison between the groups, and the trial would not have been adequately powered to detect a difference in overall survival as the primary endpoint. We also acknowledge the limitations of progression-free survival as an endpoint for pancreatic cancer—since the primary tumour has diffuse margins, accurate measurement of local progression is difficult. However, nearly 70% of the patients had new-onset metastatic disease at progression.

The trial protocol did not specify the criteria for inoperability, with this decision left to the locoregional pancreatic team (including the surgeon and specialist radiologist), in recognition of the variability in surgical practice. Most surgeons in the UK follow National Comprehensive Cancer Network guidance and we expect that this guidance was applied, although no central review of radiology took place. However, the protocol did specify that all patients had to be discussed at the multidisciplinary team meeting after chemoradiotherapy and be reassessed for operability. That few patients ultimately underwent operations suggests that the patients recruited to this trial had truly inoperable disease.

Despite its limitations, ours is the largest trial so far to report the outcome of consolidation chemoradiotherapy after a course of induction chemotherapy. The combination of radiotherapy with capecitabine was less toxic than the combination with gemcitabine, despite gemcitabine being given at a fairly low dose. This benefit was achieved with no compromise in progression-free survival and local control, and an apparent improvement in overall survival. Notwithstanding the small number of patients in this trial, the good efficacy and toxicity profile seems to favour capecitabine for combination with radiotherapy in this setting, which suggests that capecitabine could be favoured as a template regimen in trials to assess new radiosensitisers or radiotherapy dose escalation.

## References

[1] Cancer Research UK Pancreatic cancer statistics. http://www.cancerresearchuk.org/cancer-info/cancerstats/types/pancreas/?script=true.

[2] Hidalgo M (2010). Pancreatic cancer. N Engl J Med.

[3] Burris HA, Moore MJ, Andersen J (1997). Improvements in survival and clinical benefit with gemcitabine as first-line therapy for patients with advanced pancreas cancer: a randomized trial. J Clin Oncol.

[4] Gastrointestinal Tumor Study Group (1988). Treatment of locally unresectable carcinoma of the pancreas: comparison of combined-modality therapy (chemotherapy plus radiotherapy) to chemotherapy alone. J Natl Cancer Inst.

[5] Louvet C, Labianca R, Hammel P (2005). Gemcitabine in combination with oxaliplatin compared with gemcitabine alone in locally advanced or metastatic pancreatic cancer: results of a GERCOR and GISCAD phase III trial. J Clin Oncol.

[6] Moertel CG, Frytak S, Hahn RG (1981). Therapy of locally unresectable pancreatic carcinoma: a randomized comparison of high dose (6000 rads) radiation alone, moderate dose radiation (4000 rads + 5-fluorouracil), and high dose radiation + 5-fluorouracil. The gastrointestinal tumor study group. Cancer.

[7] Chauffert B, Mornex F, Bonnetain F (2008). Phase III trial comparing intensive induction chemoradiotherapy (60 Gy, infusional 5-FU and intermittent cisplatin) followed by maintenance gemcitabine with gemcitabine alone for locally advanced unresectable pancreatic cancer. Definitive results of the 2000–01 FFCD/SFRO study. Ann Oncol.

[8] Loehrer PJ, Feng Y, Cardenes H (2011). Gemcitabine alone versus gemcitabine plus radiotherapy in patients with locally advanced pancreatic cancer: an Eastern Cooperative Oncology Group trial. J Clin Oncol.

[9] Huguet F, André T, Hammel P (2007). Impact of chemoradiotherapy after disease control with chemotherapy in locally advanced pancreatic adenocarcinoma in GERCOR phase II and III studies. J Clin Oncol.

[10] Krishnan S, Rana V, Janjan NA (2007). Induction chemotherapy selects patients with locally advanced, unresectable pancreatic cancer for optimal benefit from consolidative chemoradiation therapy. Cancer.

[11] Reni M, Cereda S, Balzano G (2009). Outcome of upfront combination chemotherapy followed by chemoradiation for locally advanced pancreatic adenocarcinoma. Cancer Chemother Pharmacol.

[12] Crane CH, Varadhachary GR, Yordy JS (2011). Phase II trial of cetuximab, gemcitabine, and oxaliplatin followed by chemoradiation with cetuximab for locally advanced (T4) pancreatic adenocarcinoma: correlation of Smad4(Dpc4) immunostaining with pattern of disease progression. J Clin Oncol.

[13] Kim J-S, Lim JH, Kim JH (2012). Phase II clinical trial of induction chemotherapy with fixed dose rate gemcitabine and cisplatin followed by concurrent chemoradiotherapy with capecitabine for locally advanced pancreatic cancer. Cancer Chemother Pharmacol.

[14] Leone F, Gatti M, Massucco P (2013). Induction gemcitabine and oxaliplatin therapy followed by a twice-weekly infusion of gemcitabine and concurrent external-beam radiation for neoadjuvant treatment of locally advanced pancreatic cancer. Cancer.

[15] Ikeda M, Ioka T, Ito Y (2013). A multicenter phase II trial of S-1 with concurrent radiation therapy for locally advanced pancreatic cancer. Int J Radiat Oncol Biol Phys.

[16] Gillmore R, Laurence V, Raouf S (2010). Chemoradiotherapy with or without induction chemotherapy for locally advanced pancreatic cancer: a UK multi-institutional experience. Clin Oncol (R Coll Radiol).

[17] Sultana A, Tudur Smith C, Cunningham D, Starling N, Neoptolemos JP, Ghaneh P (2008). Meta-analyses of chemotherapy for locally advanced and metastatic pancreatic cancer: results of secondary end points analyses. Br J Cancer.

[18] Varadhachary GR, Wolff RA, Crane CH (2008). Preoperative gemcitabine and cisplatin followed by gemcitabine-based chemoradiation for resectable adenocarcinoma of the pancreatic head. J Clin Oncol.

[19] Li C-P, Chao Y, Chi K-H (2003). Concurrent chemoradiotherapy treatment of locally advanced pancreatic cancer: gemcitabine versus 5-fluorouracil, a randomized controlled study. Int J Radiat Oncol Biol Phys.

[20] Crane CH, Abbruzzese JL, Evans DB (2002). Is the therapeutic index better with gemcitabine-based chemoradiation than with 5-fluorouracil-based chemoradiation in locally advanced pancreatic cancer?. Int J Radiat Oncol Biol Phys.

[21] Wilkowski R, Boeck S, Ostermaier S (2009). Chemoradiotherapy with concurrent gemcitabine and cisplatin with or without sequential chemotherapy with gemcitabine/cisplatin vs chemoradiotherapy with concurrent 5-fluorouracil in patients with locally advanced pancreatic cancer—a multi-centre randomised phase II study. Br J Cancer.

[22] Zhu C-P, Shi J, Chen Y-X, Xie W-F, Lin Y (2011). Gemcitabine in the chemoradiotherapy for locally advanced pancreatic cancer: a meta-analysis. Radiother Oncol.

[23] Cunningham D, Chau I, Stocken DD (2009). Phase III randomized comparison of gemcitabine versus gemcitabine plus capecitabine in patients with advanced pancreatic cancer. J Clin Oncol.

[24] Aaronson NK, Ahmedzai S, Bergman B (1993). The European Organization for Research and Treatment of Cancer QLQ-C30: a quality-of-life instrument for use in international clinical trials in oncology. J Natl Cancer Inst.

[25] Chung HW, Bang SM, Park SW (2004). A prospective randomized study of gemcitabine with doxifluridine versus paclitaxel with doxifluridine in concurrent chemoradiotherapy for locally advanced pancreatic cancer. Int J Radiat Oncol Biol Phys.

[26] Brasiūnienė B, Juozaitytė E (2007). The effect of combined treatment methods on survival and toxicity in patients with pancreatic cancer. Medicina (Kaunas).

[27] Ben-Josef E, Schipper M, Francis IR (2012). A phase I/II trial of intensity modulated radiation (IMRT) dose escalation with concurrent fixed-dose rate gemcitabine (FDR-G) in patients with unresectable pancreatic cancer. Int J Radiat Oncol Biol Phys.

[28] Murphy JD, Adusumilli S, Griffith KA (2007). Full-dose gemcitabine and concurrent radiotherapy for unresectable pancreatic cancer. Int J Radiat Oncol Biol Phys.

[29] Ogawa K, Ito Y, Hirokawa N (2012). Concurrent radiotherapy and gemcitabine for unresectable pancreatic adenocarcinoma: impact of adjuvant chemotherapy on survival. Int J Radiat Oncol Biol Phys.

