# Supplementary material for: Gemcitabine-based or capecitabine-based chemoradiotherapy for locally advanced pancreatic cancer (SCALOP): a multicentre, randomised, phase 2 trial
Source: Lancet Oncol. 2013 Apr;14(4):317–26. doi: 10.1016/S1470-2045(13)70021-4 (PMC3620899; doi:10.1016/S1470-2045(13)70021-4)
Supplement: Supplementary appendix [file mmc1.pdf]

THE LANCET Oncology

Supplementary appendix

This appendix formed part of the original submission and has been peer reviewed. We post it as supplied by the authors.

Supplement to: Mukherjee S, Hurt CN, Bridgewater J, et al. Gemcitabine-based or capecitabine-based chemoradiotherapy for locally advanced pancreatic cancer (SCALOP): a multicentre, randomised, phase 2 trial. *Lancet Oncol* 2013; published online March 5. http://dx.doi.org/10.1016/S1470-2045(13)70021-4.

Appendix

Table of contents

1. Independent Trial Steering Committee (p 1)
2. Independent Data Monitoring Committee (p 1)
3. SCALOP Study Investigators (p 1)
4. Supplementary tables (p 2)
Table S1: Inclusion and exclusion criteria for the SCALOP trial
Table S2: Trial group, histology, and follow-up of the five resected patients
Table S3: QLQ-C30 global health score by treatment group

The following people participated in the study:

Independent Trial Steering Committee: Barry Hancock, Stephen Shepherd, John Wagstaff, Robert Huddard, Malcolm Mason, Jim Fitzgibbon

Independent Data Monitoring Committee: Jeff Evans, Ruth Plummer, Emma Hall

SCALOP Study Investigators:
Velindre Cancer Centre (T Crosby)
Addenbrooke's Hospital (C Jephcott)
Bristol Haematology and Oncology Centre (S Falk)
Hammersmith Hospital (H Wasan)
Northampton General Hospital (S Mukherjee)
Royal Surrey Hospital (Dr S Cummins)
Southampton General Hospital (A Bateman)
Diana Princess of Wales Hospital (Dr Roy)
Castle Hill Hospital (Dr A Maraveyas)
Queen Elizabeth Hospital, Birmingham (A Hartley, D Palmer)
Raigmore Hospital (Dr D Whillis)
Royal Free Hospital (Dr T Meyer)
Ninewells Hospital (Dr P Niblock)
North Middlesex University Hospital (J Bridgewater)
St James' University Hospital (G Radhakrishna)
Musgrove Park Hospital (J Walther)
Leicester Royal Infirmary (D Peel)
Ysbyty Gwynedd (R Neupane)
Beatson West of Scotland Oncology Centre (A Mcdonald)
Queen's Hospital, Romford (S Raouf)

Table S1: Inclusion and exclusion criteria for the SCALOP trial

Inclusion criteria

1. Age ≥ 18 years
2. Histologically or cytologically proven malignancy of the pancreas
3. Locally advanced, non-metastatic, and inoperable (or operable but medically unfit for surgery) (as discussed at MDT) malignancy. The following types of interventions are allowed:
    a. Palliative bypass procedure
    b. Common bile duct stenting
4. Primary pancreatic lesion≤7 cm in diameter as measured on a CT scan of the thorax and abdomen that has been done within four weeks before registration
5. WHO PS 0–2
6. Neutrophils ≥1·5 x 10^9/L, platelets ≥100 x 10^9/L and haemoglobin ≥10 g/dL
7. Adequate liver function tests:
    a. Serum bilirubin <35 μmol/L. In participants who have had a recent biliary drain and whose bilirubin is descending, a value of ≤50 μmol/L is acceptable
    b. AST and ALT ≤2·5 x ULN, alkaline phosphatase ≤5 x ULN
8. Adequate renal function (GFR > 50 ml/min)
9. Written informed consent obtained
10. Potentially fertile participants should agree to use an adequate contraception method, which must be continued for 12 weeks after completion of chemotherapy

Exclusion criteria

1. Women who are pregnant or breast feeding
2. Any evidence of severe uncontrolled systemic diseases including uncontrolled coronary artery disease
3. Any participant with myocardial infarction or stroke within the past six months
4. Previous malignancies in the preceding five years except for:
5. In situ cancer of the uterine cervix
6. Adequately treated basal cell skin carcinoma
7. Any early stage malignancy
8. Renal abnormalities such as adult polycystic kidney disease or hydronephrosis or ipsilateral single kidney
9. Previous RT to upper abdomen
10. Recurrent cancer following definitive pancreatic surgery
11. Lymphoma or neuroendocrine tumours of the pancreas

Table S2: Trial group, histology, and follow-up of the five resected patients

Trial group	Histology	Follow-up
Capecitabine	ypT1N0	Alive at 52 weeks
Capecitabine	ypT3N0	Alive at 52 weeks
Gemcitabine	ypT3N0	Alive at 52 weeks
Gemcitabine	ypT3N0	Alive at 52 weeks
Gemcitabine	ypT2N0	Died of postoperative complications

Table S3: QLQ-C30 global health score by treatment group

	Capecitabine chemoradiation			Gemcitabine chemoradiation		
**Week**	**Mean (SD)**	**Median (IQR)**	**% completed**	**Mean (SD)**	**Median (IQR)**	**% completed**
**0**	67·89 (3·40)	71 (50–83)	94·4	61·67 (3·60)	67 (50–75)	92·1
**17**	67·50 (3·80)	67 (58–83)	83·3	67·22 (3·04)	67 (50–75)	78·9
**23**	66·29 (4·79)	66 (50–83)	61·1	56·09 (4·49)	33 (54–75)	68·4
**26**	64·58 (4·41)	66 (54–79)	66·7	57·37 (4·24)	50 (50–83)	68·4
**39**	64·93 (4·84)	58 (50–83)	66·7	53·95 (5·27)	58 (33–67)	50·0
**52**	56·02 (6·38)	58 (42–67)	50·0	55·77 (7·69)	58 (33–83)	34·2
